# Anaerobic degradation of hexadecane and phenanthrene coupled to sulfate reduction by enriched consortia from northern Gulf of Mexico seafloor sediment

**DOI:** 10.1038/s41598-018-36567-x

**Published:** 2019-02-04

**Authors:** Boryoung Shin, Minjae Kim, Karsten Zengler, Kuk-Jeong Chin, Will A. Overholt, Lisa M. Gieg, Konstantinos T. Konstantinidis, Joel E. Kostka

**Affiliations:** 1 School of Earth and Atmospheric Sciences, Georgia Institute of Technology, Atlanta, 30332 USA; 2 School of Civil and Environmental Engineering, Georgia Institute of Technology, Atlanta, 30332 USA; 3 Department of Pediatrics, University of California, San Diego, 92093 USA; 4 Center for Microbiome Innovation, University of California, San Diego, 92093 USA; 5 Department of Biology, Georgia State University, Atlanta, 30302 USA; 6 School of Biological Sciences, Georgia Institute of Technology, Atlanta, 30332 USA; 7 Department of Biological Sciences, University of Calgary, Calgary, T2N 1N4 Canada

## Abstract

To advance understanding of the fate of hydrocarbons released from the Deepwater Horizon oil spill and deposited in marine sediments, this study characterized the microbial populations capable of anaerobic hydrocarbon degradation coupled with sulfate reduction in non-seep sediments of the northern Gulf of Mexico. Anaerobic, sediment-free enrichment cultures were obtained with either hexadecane or phenanthrene as sole carbon source and sulfate as a terminal electron acceptor. Phylogenetic analysis revealed that enriched microbial populations differed by hydrocarbon substrate, with abundant SSU rRNA gene amplicon sequences from hexadecane cultures showing high sequence identity (up to 98%) to *Desulfatibacillum alkenivorans* (family *Desulfobacteraceae*), while phenanthrene-enriched populations were most closely related to *Desulfatiglans spp*. (up to 95% sequence identity; family *Desulfarculaceae*). Assuming complete oxidation to CO_2_, observed stoichiometric ratios closely resembled the theoretical ratios of 12.25:1 for hexadecane and 8.25:1 for phenanthrene degradation coupled to sulfate reduction. Phenanthrene carboxylic acid was detected in the phenanthrene-degrading enrichment cultures, providing evidence to indicate carboxylation as an activation mechanism for phenanthrene degradation. Metagenome-assembled genomes (MAGs) revealed that phenanthrene degradation is likely mediated by novel genera or families of sulfate-reducing bacteria along with their fermentative syntrophic partners, and candidate genes linked to the degradation of aromatic hydrocarbons were detected for future study.

## Introduction

In April 2010, the Deepwater Horizon (DWH) oil rig exploded and sank, discharging approximately 4.9 million barrels of crude oil into the Gulf of Mexico (GoM) at a depth of 1544 m over the course of 86 days^1,2^. Approximately 0.5–9.1% of the released oil was deposited to the seafloor surrounding the spill site^3–5^. The seafloor surrounding the DWH wellhead is covered by fine-grained impermeable sediments that are largely anoxic below the surface 1 cm depth. Thus, a substantial amount of deposited oil was trapped in anoxic zones of marine sediments, where microbial degradation, which is a key process for ecosystem recovery from oil spill events, is understudied. Before discharged oil reaches the anoxic sediments, petroleum hydrocarbons (PHCs) are weathered and degraded by aerobic hydrocarbon-degrading bacteria^6–12^. Processing of PHCs under aerobic conditions tends to deplete labile compounds such as short-chain *n*-alkanes or simple polycyclic aromatic hydrocarbons (PAHs), leaving more complex PAHs that are resistant to degradation and may persist in sediments for long periods of time^13–17^. Once buried in the anoxic zone, microorganisms metabolize hydrocarbons through alternate electron-accepting pathways using nitrate, iron, or sulfate as their terminal electron acceptor or by fermentation^18^. Sulfate is the most abundant terminal electron acceptor present in muddy marine sediments, and therefore, anaerobic hydrocarbon degradation coupled to sulfate reduction is presumably quantitatively more important in marine sediments than degradation coupled to other electron acceptors^19^.

Biodegradation of hydrocarbons under sulfate-reducing conditions has been demonstrated for various classes of hydrocarbon compounds including saturated alkanes, BTEX compounds (Benzene, Toluene, Ethylbenzene, Xylene) and PAHs^20–26^. Whereas aliphatic alkane degradation has been studied in sulfate-reducing enrichment cultures as well as pure cultures, PAH degradation under sulfate-reducing conditions is much less understood^27–29^. To date, only one bacterial pure culture (Deltaproteobacterial strain NaphS2) has been obtained which is capable of anaerobic PAH degradation. This strain belongs to the *Desulfobacteraceae* and was isolated from a naphthalene-degrading and sulfate-reducing enrichment culture from anoxic marine sediment^30^. Mechanisms have been proposed for anaerobic naphthalene degradation coupled to sulfate reduction in studies with strain NaphS2 and an enrichment culture, N47^31–37^. The degradation pathways of all other PAHs, including phenanthrene have only been studied in enrichment cultures, as no pure cultures are available^36,38^.

Although less information is available in comparison to aerobic biodegradation pathways, some evidence suggests that anaerobic hydrocarbon degradation was enhanced in Gulf of Mexico ecosystems impacted by oil from the DWH blowout. Metabolic genes involved in both aerobic and anaerobic hydrocarbon degradation were enriched in deep-sea oil plumes generated from the spill^39^. In the deep-sea sediments close to the DWH wellhead, metagenomic analysis revealed a high potential for anaerobic hydrocarbon metabolism^40^, and sediments collected five months after the DWH discharge showed an increased abundance of genes involved in denitrification pathways^9^. In salt marsh sediments, studies reported enrichment of sulfate-reducing bacteria in parallel with Macondo oil contamination^41–44^. A novel oil-enrichment experiment with crude oil-amended flow-through sediment reactors provided quantification of the *in situ* microbial response to oil and demonstrated elevated rates of sulfate-reduction as well as methanogenesis at a natural hydrocarbon seep in the GoM^45^. Finally, anaerobic hydrocarbon degradation has been studied in hydrocarbon-rich ecosystems such as oil-contaminated sites, natural gas and oil seeps, and oil reservoirs; however, it is understudied in seafloor sediments not normally exposed to high levels of hydrocarbons^25^. Thus, the objectives of this study were (i) to characterize the microbial communities that mediate the mineralization of PHCs under sulfate-reducing conditions in anoxic sediments of the northern GoM seafloor, and more specifically (ii) to investigate the metabolic potential for anaerobic PAH degradation through a combination of metabolomics and metagenomics. The results from our highly purified enrichment cultures reveal that the predominant sulfate-reducing bacteria vary according to hydrocarbon substrate. We further provide the genomes of several novel organisms linked to respiratory and fermentative pathways during phenanthrene metabolism.

## Results

### Enrichment of hexadecane and phenanthrene-degrading microbial consortia

**Figure 1:**
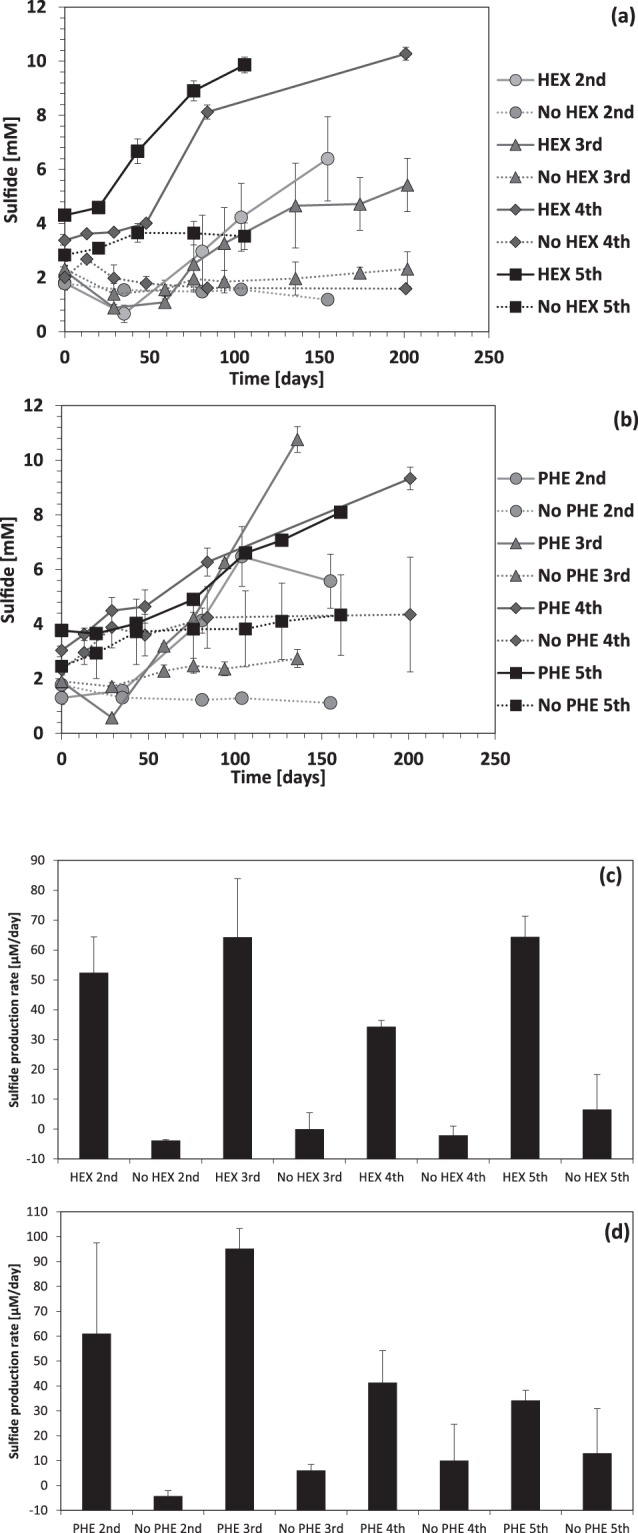
Sulfate respiration as determined by the accumulation of dissolved sulfide. (**a**) Sulfide accumulation in hexadecane-amended enrichment cultures from second, third transfers in triplicate and fourth, fifth transfers in four replicates. (**b**) Sulfide accumulation in phenanthrene-amended enrichment cultures from second, third transfers in triplicate and fourth, fifth transfers in six replicates. Solid lines depict sulfide increase with hydrocarbons, whereas dashed lines indicate stable sulfide concentration without hydrocarbons. (**c**) Sulfide production rates (μM day^−1^) calculated from logarithmic phase of bacterial activity in sulfate-reducing enrichment cultures with or without hexadecane in triplicate for second, third transfers and in four replicates for fourth, fifth transfers and (**d**) with or without phenanthrene in triplicate for second, third transfers and in four replicates for fourth, fifth transfers. Error bars indicate standard deviation in biological replicate cultures.

Samples collected from the surface 0–5 cm depth interval of northern GoM sediments were supplemented with an artificial seawater minimal medium and hexadecane or phenanthrene as a sole carbon source and electron donor. Sulfate reduction activity was monitored by the production of sulfide. Enrichment cultures were successively transferred to fresh media when sulfide concentrations reached 8–10 mM to obtain sediment-free cultures. Little to no sulfide was produced in control cultures to which no carbon substrate was added (Fig. 1a,b). The average sulfide production rate across transfers with hexadecane was 53.52 ± 16.44 µmol L^−1^ day^−1^ and 0.36 ± 7.24 µmol L^−1^ day^−1^ in the controls without hexadecane. In the phenanthrene-amended enrichment cultures, an average rate of 51.22 ± 26.69 µmol L^−1^day^−1^ sulfide production was observed, whereas 6.20 ± 12.04 µmol L^−1^day^−1^ sulfide production was observed without phenanthrene addition (Fig. 1c,d). Rates of sulfide production in phenanthrene-amended cultures equaled or exceeded those in hexadecane cultures over the first three transfers. The sulfide production rate remained stable across transfers in hexadecane-amended enrichment cultures, whereas sulfide production rate declined across transfers in phenanthrene-amended enrichment cultures. For each transfer, control cultures were created with no carbon substrate from the previously active culture. Sulfide production rate increased in the phenanthrene controls across transfers, suggesting that bacterial growth is stimulated by transferred metabolites or phenanthrene-degradation intermediates from the previous treatment.

### Microbial community structure in sediment and enrichment cultures

**Figure 2:**
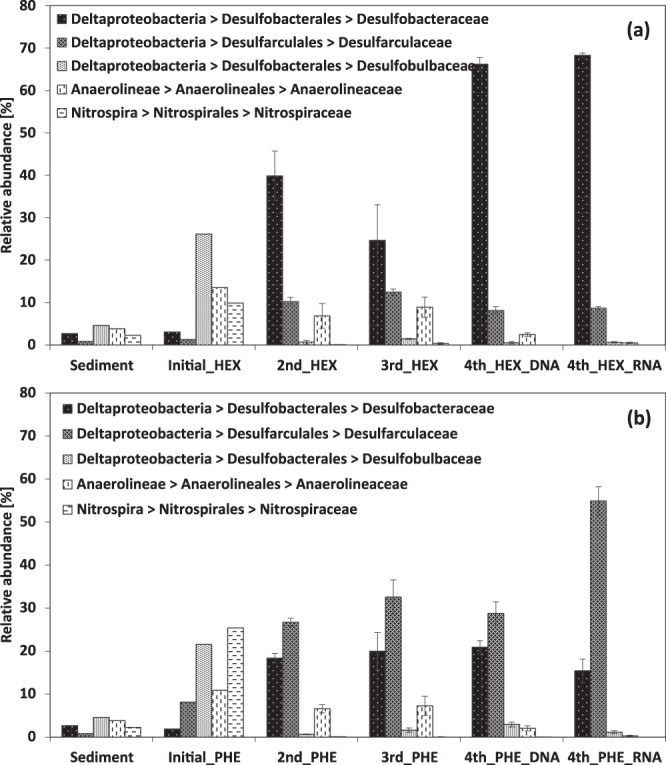
The relative abundance of microbial taxa at the family level in (**a**) hexadecane- and (**b**) phenanthrene-degrading enrichment cultures under sulfate-reducing conditions. Illumina next-generation sequencing was performed using genomic DNA isolated from various transfer stages of enrichment cultures (initial, second, third, and fourth transfers) and using total RNA isolated from fourth transfer cultures. Error bars indicate one standard deviation among biological replicates.

At the class level, *Deltaproteobacteria* constituted a relative abundance of 19.6% in the 0–5 cm depth interval from the sediment used as an inoculum, 37% in initial enrichments, and up to 86% across subsequent transfers of enrichment cultures. In contrast, members of *Nitrospira*, *Anaerolineae*, and *Gammaproteobacteria* declined in relative abundance in successive transfers (Supplemental Fig. 1). The family *Desulfobacteraceae* within the order *Desulfobacterales* comprised 2-3% relative abundance in inoculum sediment and was the most abundant group in hexadecane-amended cultures, comprising nearly 70% of the total bacterial community in the fourth transfer with similar relative abundances in the DNA- and RNA-based libraries (Fig. 2a). In the phenanthrene-degrading enrichment cultures, the family *Desulfarculaceae* within the order *Desulfarculales* comprised 0.4–1.5% relative abundance in inoculum sediment and showed the highest relative abundance throughout all transfers, especially in the RNA-based libraries where it represented 55% of the total community in the fourth transfer (Fig. 2b). The dominant detected genus within this group in the phenanthrene-degrading enrichment cultures was *Desulfatiglans*, comprising 54% of the total community within the RNA-based library. The family *Desulfobacteraceae* was the second most abundant family constituting around 21% and 15% of the total community in DNA-based and RNA-based libraries, respectively. In both hexadecane- and phenanthrene-degrading enrichment cultures, *Desulfobulbaceae*, *Anaerolineaceae*, and *Nitrospiraceae* were enriched as high as 10–26% of the total community in initial enrichment cultures but their abundance decreased over transfers (Fig. 2). In all enrichment cultures, taxonomic diversity, as determined by Shannon indices, decreased across transfers (Supplemental Fig. 2a,b). Beta diversity analysis based on the Bray-Curtis distance metric showed that the initial enrichment cultures with hexadecane or phenanthrene contained more closely related microbial communities, which then diverged based on carbon sources over successive transfers (Supplemental Fig. 2c).

### Quantitative molecular analysis of bacterial communities

In hexadecane-amended enrichment cultures, bacterial abundance as determined by qPCR of SSU rRNA genes decreased from the second (5.66 × 10^6^ copies ml^−1^) to third transfer (8.55 × 10^4^ copies ml^−1^) but increased again in the fourth transfer (5.86 × 10^6^ copies ml^−1^). In the third transfer, no difference was observed in bacterial abundance between hexadecane-amended cultures and unamended controls. In the third transfers of phenanthrene-amended cultures, we observed approximately 10 times higher bacterial abundance (2.83 × 10^6^ copies ml^−1^) in comparison to control cultures with no carbon substrate added (2.96 × 10^5^ copies ml^−1^) after 98 days of incubation. After 139 days of incubation, bacterial abundance decreased to 1.04 × 10^6^ copies ml^−1^ in phenanthrene-amended enrichment cultures, although abundance remained 10-fold higher in comparison to unamended control cultures (9.74 × 10^4^ copies ml^−1^) (Supplemental Fig. 3).

### Phylogenetic analysis of sulfate-reducing bacteria retrieved from enrichment cultures

**Figure 3:**
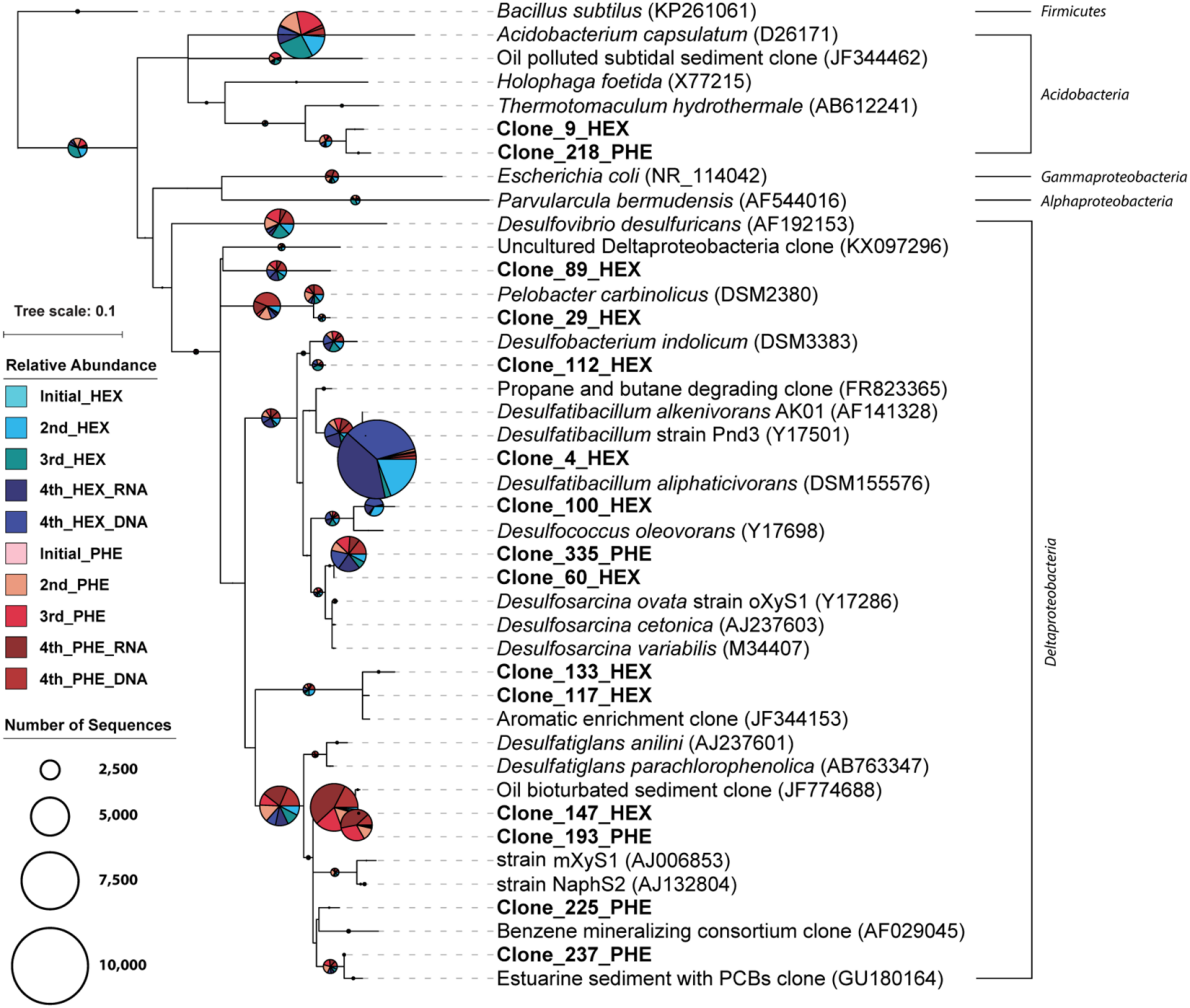
Phylogenetic analysis of SSU rRNA gene sequences retrieved from hexadecane- and phenanthrene-degrading enrichment cultures. SSU rRNA sequences from clone libraries, top isolated BLAST hits, and similar clones were used as the data set.

In order to improve phylogenetic placements and characterization of the dominant populations in the enrichment cultures, SSU rRNA gene sequences were obtained using a combination of clone library analysis and next generation sequencing. Clone libraries were screened using restriction fragment length polymorphism (RFLP) to select unique clones (data not shown). A total of 33 and 30 representative sequences (841–1525 bp) were obtained from hexadecane- and phenanthrene-degrading enrichment cultures, respectively. These sequences were inserted into the full SILVA v128 Ref_NR_99 tree using ARB parsimony. SSU rRNA gene sequences from their closest isolated relatives, previously sequenced environmental clones, sequences generated from hydrocarbon-amended cultures, as well as outgroups were included in the phylogenetic tree (Fig. 3). The most abundant clone sequence from hexadecane-amended enrichment cultures was Clone 4_HEX (GenBank accession number MG923705), which was most closely related to *Desulfatibacillum alkenivorans* strain PF2803 and *D. aliphaticivorans* strain CV2803 with sequence similarities of 98%. These *Desulfatibacillum* strains are known to degrade alkene- and/or *n*-alkane using sulfate as a terminal electron acceptor^46,47^. Clone 60_HEX (GenBank accession number MG923719) shared 96% sequence similarity with *Desulfobacterium cetonicum* strain^48^. From phenanthrene-degrading enrichment cultures, clone 193_PHE (GenBank accession number MG923739) showed the second highest relative abundance among clone sequences and was related to the aniline- and chlorophenol-degrading isolates *Desulfatiglans anilini* strain Ani1 and *Desulfatiglans parachlorophenolica* strain DS (95% sequence identity). Clone 193_PHE (GenBank accession number MG923739) sequence also shared 94% sequence identity with the anaerobic naphthalene-degrading pure culture strain NaphS2^30^.

### Degradation of hexadecane and phenanthrene coupled to sulfate reduction

Mineralization of hexadecane or phenanthrene in enrichment cultures was demonstrated by quantifying hexadecane or phenanthrene loss in the 2,2,4,4,6,8,8-heptamethylnonane (HMN) layer and sulfate loss in the aqueous phase (Table 1). Observed stoichiometric ratios were close to the theoretical ratios of 12.25:1 for hexadecane and 8.25:1 for phenanthrene degradation coupled to sulfate reduction.

**Table 1:** Calculated electron balances for enrichment cultures grown on hexadecane or phenanthrene as electron donor and sulfate as electron acceptor.

Hexadecane degradation C_16_H_34_ + 12.25SO_4_^2-^ + 8.5H^+^ → 16HCO_3_^−^ + 12.25H_2_S + H_2_O				
Incubation time (day)	Hexadecane loss (μmole)	Expected sulfate loss (μmole)	Observed sulfate loss (μmole)	% of expected
230 (4^th^ transfer)	34.78 ± 3.83	426.05	568.09 ± 44.40	133.34
121 (5^th^ transfer)	29.67 ± 3.66	484.59	473.82 ± 55.60	97.78
**Phenanthrene degradation** **C**_**14**_**H**_**10**_** + 8.25 SO**_**4**_^**2−**^** + 9H**_**2**_**O + 2.5H**^**+**^ **→ 14HCO**_**3**_^−^** + 8.25H**_**2**_**S**				
**Incubation time** **(day)**	**Phenanthrene loss (μmole)**	**Expected sulfate loss (μmole)**	**Observed sulfate loss (μmole)**	**% of expected**
230 (4^th^ transfer)	35.83 ± 7.2	295.62	365.13 ± 49.64	123.51

**Figure 4:**
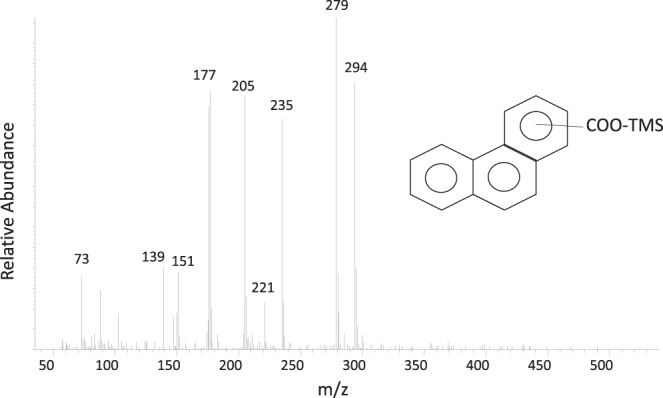
Mass spectrum of a tentatively identified phenanthrene carboxylic acid (shown as its trimethylsilyl (TMS) derivative). While the isomer is unknown, the mass spectral profile matches closely with that published by Davidova *et al*.^38^.

Metabolite analysis using GC-MS (gas chromatography-mass spectrometry) from the phenanthrene cultures revealed the presence of a putative metabolite that was identified as phenanthrene carboxylic acid (Fig. 4). The identified metabolite had a mass spectral profile identical to that of phenanthrene-2-carboxylic acid which was positively identified in a different sulfate-reducing phenanthrene-degrading enrichment^38^. Another potential metabolite identified as hydroxybenzoic acid was also detected (Supplemental Fig. 4). Based on comparisons with calibration curves, phenanthrene carboxylic acid was detected in the enrichment at a concentration of 1 μM, while hydroxybenzoic acid was detected at 3.8 μM.

### Metagenomic analysis of phenanthrene-degrading enrichment cultures

Due to the paucity of information available on anaerobic degradation of polycyclic aromatic hydrocarbons (PAHs), metagenomic analysis was performed on the 4^th^ transfer of the phenanthrene enrichment cultures (PHE 4^th^). Three replicate metagenomes were obtained, with 4 to 7 million reads each after trimming (paired-end reads with average length of 170–196 bp per dataset). The estimated abundance-weighted average coverage estimated by Nonpareil^49^ for the three metagenomes ranged between 77–82% (Supplemental Fig. 5), indicating a medium-to-low community complexity and sufficient coverage for assembly and genome binning. Nonpareil curves also indicated that the communities in three replicates of PHE 4th transfers showed a similar sequence complexity among themselves (Supplemental Fig. 5). Metagenomes were co-assembled into 101,806 contigs with N50 of 3,035 bp. A total of 9 good quality MAGs were recovered from the co-assembly, and whole genome comparisons to available genomes using the Microbial Genomes Atlas (MiGA) webserver^50^ revealed that the genome MAGs represent taxa at the order or class level most closely related to members of the *Bacterioidales*, *Deltaproteobacteria*, *Desulfobacterales* and *Desulfovibrionales*. The only exception to this was Phe_bin_3, which was closely related to a described species, *Pelobacter carbinolicus* (Table 2). Pairwise comparisons of the 9 genome MAGs showed a range of 36–52% genome-aggregate amino acid identity (AAI) (Supplemental Fig. 6), which suggested none of the genome MAGs are close relatives of each other. The relative abundance of the 9 MAGs summed to 85–91% of the total metagenome from triplicate cultures, consistent with the SSU rRNA gene results reported above. Specifically, *Bacteroidales* Phe_bin_001 and *Deltaproteobacteria* Phe_bin_002 together represented more than 50% of the communities (Supplemental Table 1). Results indicate that these two genome MAGs likely represented key players in phenanthrene degradation.

**Table 2:** Taxonomic classification of genome bins recovered from the PHE 4^th^ transfer metagenomes.

Bin_ID	Taxonomic classification		Closest relative	Amino acid identity (AAI)
Phe_bin_001	class Bacteroidia****	order Bacteroidales*	Draconibacterium_orientale_NZ_CP007451	48.15
Phe_bin_002	phylum Proteobacteria****	class Deltaproteobacteria*	Desulfococcus_multivorans_NZ_CP015381	43.61
Phe_bin_003	genus Pelobacter****	species Pelobacter carbinolicus***	Pelobacter_carbinolicus_DSM_2380_NC_007498	91.2
Phe_bin_004	phylum Proteobacteria****	class Deltaproteobacteria**	Pelobacter_carbinolicus_DSM_2380_NC_007498	44.47
Phe_bin_005	phylum Proteobacteria***	class Deltaproteobacteria*	Pelobacter_acetylenicus_NZ_CP015455	40.16
Phe_bin_006	class Deltaproteobacteria****	order Desulfobacterales*	Desulfococcus_multivorans_NZ_CP015381	51.24
Phe_bin_026	class Deltaproteobacteria****	order Desulfobacterales*	Desulfococcus_multivorans_NZ_CP015381	48.14
Phe_bin_030	phylum Proteobacteria**	class Betaproteobacteria*	Candidatus_Tremblaya_princeps_LN998830	39.6
Phe_bin_031	class Deltaproteobacteria****	order Desulfovibrionales*	Desulfovibrio_salexigens_DSM_2638_NC_012881	53.8

Significance at p-value below: *0.5, **0.1, ***0.05, ****0.01.

Metagenome sequencing showed that several of the recovered MAGs harbor genes that are linked to the degradation of monoaromatic compounds, and potentially PAHs. Protein annotation of high-quality MAGs revealed that Phe_bin_001 and Phe_bin_002 contained genes involved in salicylate and gentisate catabolism such as salicylate esterase and fumarylacetoacetate hydrolase. Specifically, the genome of Phe_bin_001 also encodes genes involved in the homogentisate pathway of aromatic compound degradation. Salicylate (2-hydroxybenzoate) is formed during the degradation of phenolic compounds. For example, *Desulfobacterium cetonicum* was previously shown to oxidize *m*-cresol to 3-hydroxybenzoate by fumarate addition to the methyl group^51^, while *Desulfobacterium aniline* strain AK1 was shown to degrade phenol via phosphorylation to phenylphosphate, followed by carboxylation to 4-hydroxybenzoate^52^.

In particular, Phe_bin_002 showed a high potential to degrade aromatic compounds. For example, a gene from bin 002 (contig_100_493_6) matched with the gene encoding (S)-1-Phenylethanol dehydrogenase which was shown to mediate the anaerobic degradation of ethylbenzene^53^. In addition, genes encoding acetophenone carboxylase gamma, beta, delta subunits, which have been implicated in the carboxylation of acetophenone by *Aromatoleum aromaticum*, were detected in bin 002 (from contig_100_1186_4 to contig_100_1185_8) with good amino acid identities and coverage^54^. Also, Phe_bin_002 contained anaerobic 4-hydroxybenzoate carboxylase (bsdBC/UbiX), which suggests the potential for hydroxybenzoate oxidation, in agreement with our metabolite analysis. Previously, it was shown that UbiX-like carboxylase (3-octaprenyl-4-hydroxybenzoate carboxylase) was specifically expressed during benzene degradation in an iron-reducing enrichment culture^55^. The genome of *Desulfatiglans aniline* strain Anil1 identified two gene clusters containing genes encoding UbiD-like proteins and a UbiX-like protein. Genes involved in aryl-alcohol dehydrogenase (*adhCP*), alcohol dehydrogenase (*frmA*, *yiaY*, ADH5), and acetaldehyde dehydrogenase (*adhE*) were detected in the majority of MAGs, indicating that the enrichment culture has the potential to degrade monoaromatic compounds such as toluene, xylene, methylnaphthalene, cyclohexanol, and phenol. Although we could not confirm genes encoding for phenanthrene degradation, we provide potential genetic targets for further experimental verification.

A complete set of dissimilatory sulfate reduction and oxidation genes such as dissimilatory sulfite reductase alpha and beta subunits (*dsrAB*), adenylsulfate reductase subunit AB (*aprAB*), and sulfate adenylyltransferase were recovered from the MAGs (Supplemental Table 2).

## Discussion

Anaerobic microorganisms that degrade hydrocarbons are understudied relative to their aerobic counterparts and our knowledge of anaerobic PAH degradation is still in its infancy^18^. A number of strains have been isolated and their metabolic pathways characterized for anaerobic alkane degradation, whereas very little information is available on the biochemical mechanisms of anaerobic PAH degradation. In the case of both alkanes and PAHs, the ecology of anaerobes that degrade them in the environment remains unclear. Phenanthrene represents the highest molecular weight PAH compound which was shown to be degraded under anoxic conditions^18^, and this compound is therefore likely to persist of long periods after oil discharge into the environment. Since PAHs are carcinogenic, mutagenic, and toxic to organisms, microbial activation and metabolism is an essential component of removing persistent PAHs from the environment^56^. Thus, we chose to investigate the microbial populations that couple degradation of representative alkane (hexadecane) and PAH (phenanthrene) compounds to sulfate reduction, which is the dominant anaerobic terminal electron accepting process in marine sediments.

Chemical analysis of sulfate and petroleum hydrocarbons confirmed the activity of our highly purified sediment-free enrichments. The consumption of hexadecane or phenanthrene was stoichiometric with sulfate depletion, in corroboration of previous studies. So and Young observed a loss of sulfate that corresponded to 89% in comparison to the expected stoichiometry calculated from hexadecane consumption in their enrichment cultures^57^. In this study, 98% and 133% predicted sulfate loss was observed based on hexadecane consumption. Sulfate reduction rates in the So and Young study were 6 times higher (approximately 315 µmol L^−1^ day^−1^) than those observed in this study (53.21 ± 16.44 µmol L^−1^ day^−1^); however, this may be explained by the fact that So and Young used a 3 times higher initial hexadecane concentration. In phenanthrene amended enrichment cultures, Davidova *et al*. observed 109% of predicted sulfate loss based on consumption of carbon substrate^38^, whereas we observed a 124% sulfate loss calculated from phenanthrene consumption. Higher sulfate depletion in our study may be explained by sulfate reduction coupled with the oxidation of intermediates of hydrocarbon metabolism carried over from previous culture transfers. These results confirm that hexadecane and phenanthrene degradation are closely coupled to sulfate reduction in enrichment cultures from this study. Further, the identification of phenanthrene carboxylic acid, a previously-reported metabolite from sulfidogenic phenanthrene biodegradation^36,38^, suggests carboxylation as a mode of phenanthrene activation in the present culture, though additional studies are needed to elucidate the phenanthrene biodegradation pathway.

This is the first cultivation-based study that elucidates the anaerobic microbial populations which degrade alkanes or PAHs in GoM sediments distant from natural seeps where the indigenous microbial communities are not as likely to be conditioned or primed to degrade hydrocarbons^58^. The most abundant microbial group in the GoM sediments studied here was *Desulfobulbus* within the family *Desulfobulbaceae*, comprising 3.9% of the total microbial community. The relative abundance of *Desulfobulbaceae* increased in initial enrichment cultures to as high as 21–26% but it decreased to 1-2% after successive transfers in both hexadecane- and phenanthrene-degrading enrichments. Cultured members of the *Desulfobulbaceae* generally use short-chain fatty acids as carbon substrates, which suggests that their abundance increased when carbon substrates from inoculum sediment were available and decreased over successive transfers^59^. All enrichment cultures were dominated by *Deltaproteobacteria*, which rose to near 90% of all SSU rRNA gene sequences retrieved in comparison to a relative abundance of 17–21% in the sediment inoculum. However, within the *Deltaproteobacteria*, the dominant microbial populations in the phenanthrene-degrading enrichments were distinct from those of hexadecane-degrading enrichment cultures. Whereas dominant populations closely related to known alkane-degrading *Desulfatibacillum* strains in the *Desulfobacteraceae* were detected in cultures grown on hexadecane, populations more closely related to *Desulfatiglans* of the *Desulfarculaceae* predominated when phenanthrene was the sole carbon and energy source. *Desulfobacteraceae* and *Desulfarculaceae* comprised 2-3% and 0.5–1.5% of the total community in the sediment used as an inoculum, respectively, and their relative abundance increased up to 67% and 55% in hexadecane- and phenanthrene-amended enrichment cultures. Results from RNA-based SSU rRNA amplicon libraries showed that these families of sulfate-reducers are not only abundant but represent the most active members in the enrichment cultures (Fig. 2).

The results presented here are corroborated by previous work on sulfate-reducing bacteria capable of alkane degradation^25,60^. For example, close relatives of SSU rRNA clone sequences retrieved from our cultures show high sequence identity to known alkane-degraders such as *Desulfatibacillum alkenivorans* AK-01 and *Desulfatibacillum aliphaticivorans* CV2803. *Desulfatibacillum alkenivorans* AK-01 was isolated from petroleum-contaminated sediment collected from the Arthur Kill estuary in New York and was shown to be capable of degradation of C_13_-C_18_ alkanes, C_15_-C_16_ alkenes, and C_15_-C_16_ alkanols^61^. In addition, members of the *Desulfosarcina* and *Desulfococcus* clades, which were detected in our hexadecane enrichments, were previously identified as key alkane-degraders in sediments of natural hydrocarbon seeps^62^.

Despite a paucity of information on PAH degradation under sulfate-reducing conditions, we observed some similarities to previous studies of oil-contaminated marine sediments and pure cultures of sulfate-reducers grown on monoaromatic compounds. The majority of clone sequences from phenanthrene-degrading enrichment cultures in this study were affiliated to the genus *Desulfatiglans* within the family *Desulfarculaceae* and were highly similar (>97% sequence identity) to Illumina MiSeq amplicon sequences that constituted 55% of the RNA-based total community. This indicates that *Desulfatiglans* spp. may play a key role in phenanthrene degradation under sulfate-reducing conditions. Characterized isolates which show high sequence identity (95%) to sequences retrieved from our enrichment cultures include *Desulfatiglans anilini* strain Ani1, *Desulfatiglans parachlorophenolica* strain DS, and *Desulfosarcina ovata* strain oXyS1, that are all known to metabolize monoaromatic compounds (aniline-, parachlorophenol-, and *o*-xylene)^63–65^. The enrichment of *Desulfarculaceae* from sediments in the northern GoM is consistent with results from oil-polluted subtidal sediments investigated on the Spanish coast after the Prestige oil spill^66^. In contrast, clone sequences retrieved from a phenanthrene-degrading enrichment of hydrocarbon-contaminated marine sediments in San Diego Bay^38^ were not closely related to clone sequences from this study.

Little information is available on phenanthrene degradation under sulfate-reducing conditions^36,38,67^. A number of PAH compounds, including those containing >4 rings (such as phenanthrene), were shown to be degraded in previous studies of sulfate-reducing enrichment cultures^18^. However, only PAH compounds containing 2 or 3 rings were shown to be utilized as the sole carbon and energy source. The majority of results and the only pure culture were obtained on 2 ring naphthalene. Sulfate-reducing strain NaphS2 and the enrichment N47 were both shown to degrade naphthalene and 2-methyl-napthalene. *Deltaproteobacteria* in these cultures are only distantly related to each other and the enzymes involved in their degradation remain unknown^18^.

Overall, our results showed that genera of *Desulfococcus*, *Desulfatibacillum*, and *Desulfosarcina* may be the key players in *n*-alkane degradation, whereas *Desulfatiglans* spp. is linked to PAH degradation under sulfate-reducing conditions.

Another possible mechanism for hexadecane and phenanthrene degradation in enrichment cultures in this study is syntrophic biodegradation^68^. We suggest that hexadecane or phenanthrene may have been mineralized by the coupled mutualistic interaction between hydrocarbon-fermenting and H_2_/acetate/formate-utilizing microorganisms. Previous studies have proposed that members of the *Pelotomaculum*, *Pelobacter*, and *Syntrophaceae* groups carry out hydrocarbon fermentation in syntrophic consortia that mineralize hydrocarbons under sulfate-reducing conditions^69–73^. In this study, the putative hydrocarbon fermenter, *Pelobacter*, was detected in abundance in both hexadecane- and phenanthrene-degrading enrichment cultures (0.4–3.1% and 1.8–10.4% relative abundance, respectively), in corroboration of previous work. Members of the *Syntrophaceae* were also detected in all enrichment cultures (0.1–1% relative abundance). These microbial groups could produce intermediates such as H_2_ and/or acetate that are supplied to sulfate-reducing bacteria in a syntrophic relationship. The *Desulfobacteraceae* have been implicated as hydrogenotrophs in hexadecane-degrading enrichment cultures that consume hydrogen produced from initial fermentation of hydrocarbons^70^. In phenanthrene-degrading enrichment cultures, the family *Desulfarculaceae* may play a role as a formate and/or acetate utilizing microbial group. The only isolates within the family *Desulfarculaceae, Desulfarculus baarsii* and *Desulfatiglans anilini* strain DS, were shown to oxidize formate, acetate, butyrate, pyruvate, other short-chain and long-chain fatty acids to CO_2_^64,74^. Potential secondary fermenters or scavengers such as *Ignavibacteria* and *Anaerolineae* were also detected in all enrichment cultures but their relative abundance decreased over transfers^69^. However, syntrophic degradation of hexadecane and phenanthrene in enrichment cultures from this study requires further confirmation.

In general, metagenomic analysis of microbial communities in phenanthrene-degrading cultures corroborated characterization based on SSU rRNA gene amplicon sequencing at a broad taxonomic level. The same families of the *Deltaproteobacteria* were detected in high proportions in the metagenome and amplicon libraries (Supplemental Figs 1, 7, 8 and Supplemental Table 1). Assembly and binning of the metagenomes revealed 9 genome MAGs, the majority of which appear to represent a novel order or class. Representing over 50% of metagenomes detected, MAGs 001 and 002 appear to represent organisms closely linked to phenanthrene metabolism. Of these, only bin 002, which represents 30% of the metagenome, contained a complete set of genes for dissimilatory sulfate reduction. Based on blastn analysis of the *dsrAB* genes retrieved from bin 002 using the fungene database, bin 002 shared the highest sequence similarity (~82%) to sulfate-reducing strain mXyS1 (known to degrade xylene), which contains an SSU rRNA gene that is closely related to *Desulfosarcina variabilis*. Since *Desulfatiglans* showed the highest relative abundance in our amplicon data, we attempted to recruit our metagenome reads to the genome of *Desulfatiglans anilini* DSM 4660, the only sequenced genome available for this genus. Although this analysis revealed that there may be some organisms related to *Desulfatiglans* in our cultures, the results indicated that the assembled MAGs did not represent closely related organisms. Thus, we conclude that the dominant microbial populations present in our phenanthrene cultures represent new members of the *Deltaproteobacteria* that are not closely related to characterized organisms.

Although this study was not able to definitively identify genes encoding phenanthrene degradation proteins, we did identify genes linked to the degradation of aromatic compounds, which can be employed as genetic targets in future studies of phenanthrene degradation. Further, genes for the degradation of some intermediates of PAH degradation that were previously reported from anaerobic consortia were also identified in the recovered MAGs. We suggest that a metagenomic/metatranscriptomic study of all existing sulfate-reducing, PAH degrading cultures will likely provide novel information on the organisms and metabolic pathways involved in phenanthrene degradation.

In summary, our understanding of the biochemical pathways of anaerobic hydrocarbon degradation is a key knowledge gap for predicting the long-term fate of recalcitrant oil compounds in fine-grained sediments that cover much of the seafloor. Here we couple cultivation with metagenomics and metabolomics to uncover the dominant microbial populations and their metabolic potential in enriched sulfate-reducing consortia capable of the mineralization of hexadecane and phenanthrene. The results revealed the taxonomy and metabolic potential of sulfate-reducing bacteria linked to anaerobic hexadecane and phenanthrene degradation. The degradation pathways of these hydrocarbon compounds is likely mediated by novel sulfate-reducing bacteria as well as syntrophic partners. To our knowledge, this is the first characterization of anaerobic hydrocarbon-degrading microbial populations in non-seep marine sediments that were not pre-exposed to extensive hydrocarbon inputs in the GoM. This is significant since most of the seafloor is not exposed to high levels of petroleum hydrocarbons prior to a spill. In addition, we have identified key taxa that may be used as model organisms in conceptual models for the natural attenuation of oil contamination in anoxic marine muds.

## Methods

### Sediment sample collection

Sediment samples were collected from the northern Gulf of Mexico (29.3989 N, –88.8678 W) during an expedition with the R/V Weatherbird on August 21 in 2013, 87 kilometers NW from Macondo wellhead near the Mississippi river Delta at 56 m water depth. To the best of our knowledge, the study site was not exposed to oil contamination from the Deepwater Horizon oil spill in 2010^5^. A sediment core sample was sectioned on board at 2 mm and 5 mm intervals from 0–2 cm and 2–10 cm sediment depth, respectively. Surface (0–5 cm depth) sediment core samples were collected in sterile plastic bags for cultivation and immediately stored at 4 °C for 2 months until enrichment cultures were initiated. Parallel samples were frozen immediately for amplicon sequencing. At this site, oxygen is depleted within the top few millimeters of the sediment surface and the collected samples from the anoxic zone.

### Enrichment culture setup

Sediment from the 0–5 cm depth interval was homogenized and supplemented as a 10% (w/v) inoculum with sterile anaerobic artificial seawater medium (composition per liter: 20.0 g NaCl, 3.0 g MgCl_2_∙6H_2_O, 0.15 g CaCl_2_∙2H_2_O, 0.3 g NH_4_Cl, 0.2 g KH_2_PO_4_, 0.5 g KCl, 4.0 g Na_2_SO_4_, 1 ml trace elements A, 1 ml trace elements B, 1 ml vitamin mixture, 1 ml thiamine solution, 1 ml B_12_ solution, and buffered with 30 mM NaHCO_3_; modified from^19^. The medium was amended with 1 mM of sodium sulfide as a reducing agent and 0.0001% solution of resazurin as a redox indicator. Triplicate enrichment cultures were amended with either hexadecane (99%, Acros Organics, Morris Plains, NJ) or phenanthrene (98%, St. Louis, MO) as a sole carbon source in HMN (hexadecane; 11.32 g per liter HMN, phenanthrene; 8.91 g L per liter HMN) as the inert carrier (98%, Acros Organics, Morris Plains, NJ) along with hydrocarbon-free controls (4 ml HMN per bottle). One hundred ml of enrichment culture was prepared in 165-ml serum bottles with a N_2_/CO_2_ (90:10 v/v) headspace and sealed with butyl rubber stoppers. Strictly anaerobic technique was used throughout all steps for enrichment culture preparation. The bottles were incubated horizontally to maximize contact between the medium and hydrocarbon layer and to minimize contact between HMN layer and butyl rubber stopper. Enrichment cultures were incubated at 30 °C in the dark without agitation. Sulfate reduction activity was monitored by measuring the accumulation of sulfide using the methylene blue method^75^. When the sulfide concentration reached ~8 mM, enrichment cultures were successively transferred five times into fresh medium (20% v/v) to obtain sediment-free cultures.

### Nucleic acid extraction and analysis of SSU rRNA sequences

Total genomic DNA was extracted from parallel frozen samples of the same 0–5 cm depth interval as that used for cultivation, and 10 ml of enrichment cultures from the first, third, and fourth generations using a MoBio PowerSoil DNA isolation kit (MoBio Laboratories, Carlsbad, CA) with slight modifications from the manufacturer’s protocol as follows. Ten ml of each enrichment culture was centrifuged at 10,000 × g for 5 minutes in sterile falcon tubes and the resulting cell pellet was transferred to the provided 2 ml bead tube. Total RNA was extracted using the Direct-zol RNA miniPrep kit (Zymo Research, Irvine, CA) with slight modifications as follows: Forty ml of the fourth generation sediment-free enrichment cultures were centrifuged at 10,000 × g for 5 minutes. Total RNA from cell pellets was stabilized by adding 1 ml of TRI Reagent (Zymo Research, Irvine, CA) and incubated at room temperature for 5 min. A 200 μl aliquot of cold chloroform was added, incubated at room temperature for 3 min, and shaken vigorously for 15 seconds. Total RNA was extracted from the aqueous phase following the manufacturer’s protocol. A 10 μl aliquot of total RNA was separated by agarose gel electrophoresis to assess RNA quality^76^. Total RNA was reverse transcribed to DNA using qScript XLT cDNA SuperMix (Quanta Biosciences, Beverly, MA) according to the manufacturer’s protocol. For Illumina sequencing, PCR amplification was performed using 515F and 806R primers from both DNA and cDNA as described by the Earth Microbiome Project (http://www.earthmicrobiome.org/emp- standard-protocols/dna-extraction-protocol/)^77,78^. PCR products were barcoded using an Access Array Barcode Library (Fluidigm, South San Francisco, CA), purified using an E.Z.N.A Cycle Pure Kit (Omega Bio-tek, Norcross, GA), and pooled together based on DNA concentration. Purified and pooled PCR amplicons were sequenced using an Illumina MiSeq platform (Illumina, San Diego, CA). Sequence analysis was accomplished using the software QIIME ver. 1.9.1^77^ and Mothur ver. 1.38.0^79^. Sequences with a quality score below 20 were removed using Mothur ver. 1.38.0 and clustered into operational taxonomic units (OTUs) by 97% sequence identity using UCLUST^80^ implemented in QIIME ver. 1.9.1. Representative sequences were aligned against the SILVA ver. 123 database (https://www.arb-silva.de/) and chimeric sequences were removed using UCHIME^80^ implemented in Mothur ver. 1.38.0. Taxonomy was assigned using the RDP classification algorithm set at a 50% confidence rating with the SILVA Small Subunit rRNA Database release 123 (https://www.arb-silva.de/no_cache/download/archive/release_123/Exports/). The resultant OTU table was normalized using the CSS algorithm implemented in QIIME ver. 1.9.1^81^. Shannon index was calculated with QIIME ver. 1.9.1. A Bray-Curtis distance matrix was obtained from the rarefied OTU table and used to generate a principal coordinate analysis (PCoA) plot.

### Quantitative molecular analyses for total bacteria

In order to quantify total bacterial SSU rRNA gene abundance, quantitative real-time PCR (qPCR) was performed using the total DNA extracted from either hexadecane- or phenanthrene-amended enrichment cultures from second, third, and fourth transfers as a template. Reactions were performed with PowerUp SYBR Green Mastermix (Applied Biosystems, Foster City, CA) using the primers 331 F (5′-TCC TAC GGG AGG CAG CAG T-3′) and 515 R (5′-ATT ACC GCG GCT GCT GG-3′) targeting bacterial SSU rRNA genes on a StepOne Plus Real-Time PCR System (Applied Biosystems, Foster City, CA). The following qPCR program was used: 50 °C for 2 min, 95 °C for 2 min; 95 °C for 15 sec, 55 °C for 15 sec, 72 °C for 1 min (40 cycles) followed by melt curve analysis with a temperature gradient from 60 °C to 95 °C at an increment of 0.3 °C/min. All reactions were performed in technical triplicate and analyzed using StepOne Software v. 2.3. A standard curve with an efficiency of 102.96 ± 0.27% was generated by serial dilution of pGEM-T Easy Vector plasmids (Promega, Madison, WI) containing the full-length *E. coli* SSU rRNA gene and used for absolute quantification.

### Cloning and Sanger sequencing

Due to uncertainties in taxonomic affiliation and the phylogenetic placement of dominant populations from the 250 bp Illumina sequences, PCR was performed in order to obtain longer (close to full length) 16S SSU rRNA gene sequences using 27F/1492R primers (Lane, 1991) with DNA extracted from third generation enrichment cultures. PCR products were purified using an E.Z.N.A Cycle Pure Kit (Omega Bio-tek, Norcross, GA) and cloned into pGEM-T Easy Vector (Promega, Madison, WI). Vectors were transformed into DH5α *E. coli* competent cells (Invitrogen, Carlsbad, CA) and 344 positive colonies were selected by blue/white screening. PCR amplification was performed from colonies using vector-specific primers - T7P (5′-TAA TAC GAC TCA CTA TAG GG-3′) and SP6 (5′-ATT TAG GTG ACA CTA TAG AA-3′) - and run on a 1% (w/v) agarose gel to confirm full-length 16S SSU rRNA gene insertion. PCR products were purified using an E.Z.N.A Cycle Pure Kit (Omega Bio-tek, Norcross, GA) and digested by the HhaI restriction enzyme (New England Biolabs, Ipswich, MA). Clones were grouped according to their restriction fragment length polymorphism (RFLP) patterns and 78 representative clones were selected. Representative clones were incubated at 37 °C in liquid LB media (Amresco, Solon, OH) and plasmids were extracted using a GET Plasmid Mini Prep kit following the manufacturer’s manual (G-Biosciences, Louis, MO). Purified plasmids were sent to the Eurofins Genomics facility in Tucker, GA, USA for Sanger sequencing and sequencing was performed in 2 directions.

All SSU rRNA gene sequences from the clone libraries were aligned using PyNAST trained on the SILVA seed database v.128 available in Mothur and inserted into a pre-made ARB tree (SILVA v.128 Ref_NR_99) using the parsimony function implemented in ARB^82–85^. Close relatives to clone sequences were manually chosen as well as outgroups to anchor the tree structure. All Illumina-derived OTU references sequences were also aligned to the SILVA seed database and inserted into the reference tree using RAxML v.8.2.10 with the environment placement algorithm^86^. The resulting JPlace file was modified using a custom script as well as the JPlace.to_iTol.rb script that can be found in the enveomics collection^87^. The tree was visualized using the interactive tree of life^88^. More details can be found at http://waoverholt.github.io/RaXML-EPA-iToL, including all commands and scripts used.

### Determination of loss of hexadecane and phenanthrene coupled to sulfate reduction

Subsamples of enrichment cultures were used to measure sulfate concentrations in media and duplicates of whole cultures were sacrificed for hydrocarbon analysis. Sulfate concentrations were determined by high-performance liquid chromatography (HPLC) analysis with a Waters 1525 binary high-pressure pump coupled with a absorbance detector Waters 2487 Dual UV-vis^89^. Sulfate losses relative to carbon substrate-unamended controls were used for stoichiometric calculations. To demonstrate hexadecane and phenanthrene degradation, total hydrocarbons were extracted from enrichment cultures. In brief, three extractions of 25 ml of dichloromethane (DCM) each were performed and the resulting organic layers were collected using separatory funnels. Extracts were dried by filtering through sodium sulfate and glass wool and subsequently evaporated using a TurboVapII (Biotage, Uppsala, Sweden) under flow of N_2_ gas at 36 °C. Subsamples of 1 μl from the extracts were analyzed by gas chromatography with flame ionization detection (GC-2014, Shimadzu, Kyoto, Japan) using a Rxi-5Sil MS column (30 m length, 0.32 mm internal diameter) (Restek, Bellefonte, PA). Hydrogen was used as carrier gas and the injector temperature was 315 °C. The column temperature was initially set at 60 °C and increased by 8 °C min^−1^ to 290 °C. The total loss of hexadecane or phenanthrene was calculated by comparing with uninoculated controls.

### Metabolite analysis

Once phenanthrene degradation was demonstrated, replicate incubations were sacrificed for metabolite analysis after 150 days of incubation from the 4^th^ transfer. Two 50-mL cultures were acidified to pH 2, then extracted separately with 3 aliquots of ethyl acetate. Organic extracts were combined and concentrated to 50 μL, then silylated by adding 50 μL N-, O-bistrimethylsilylacetamide. The sample was analyzed on an gas chromatograph (Agilent 7890 A) coupled with a mass spectrometer (Agilent 5975 C) equipped with a 50 m HP1-MS column as previously described^90^. The concentration of the tentatively identified phenanthrene carboxylic acid (not commercially-available) was estimated using a calibration curve prepared from an authentic standard of 2-naphthoic acid, while the putative hydroxybenzoic acid was quantified using authentic 4-hydroxybenzoic acid.

### Metagenome shotgun sequencing and sequence analysis

Total genomic DNA was sequenced at the Georgia Institute of Technology sequencing facility on both Illumina HiSeq and MiSeq platforms. The DNA was fragmented and the library was prepared using TruSeq Kit (Illumina) according to the manufacturer’s protocol. Triplicates of total DNA extracts from enrichment cultures were sequenced in one flow cell lane with a 2 × 150 bp and 2 × 250 bp paired-end format for the HiSeq and MiSeq platforms, respectively. Raw Illumina reads were trimmed using a Q = 20 PHRED quality score and 50 bp length cutoff using SolexaQA^91^ for further analysis. Nonpareil version 3.20 was used with default parameters to estimate the level of coverage of the community by sequencing on only one of the paired reads as recommended previously^49,92^. Trimmed reads from three replicates were merged using PEAR^93^. All merged and unmerged reads combined for co-assembly with IDBA-UD^94^ (default options). Resulting contigs were binned using MaxBin. CheckM was used to estimate completeness and contamination of each bin^95,96^. Quality of MAGs was calculated (Quality = Completeness − 5 × Contamination) and MAGs with a quality score above 50 were used for further analysis. MAGs with good quality were uploaded on the Rapid Annotation using Subsystem Technology (http://rast.nmpdr.org/) and BlastKORALA and GhostKOALA on Kyoto Encyclopedia of Genes and Genomes (KEGG) for functional annotation. Microbial Genome Atlas (MiGA) webserver was used to determine the most likely taxonomic classification and novelty rank from good quality MAGs against the classified species in NCBI’s prokaryotic genome database (available at www.microbial-genomes.org). Read recruitment plots were obtained as described previously with a minimum cut-off for a match of 70% of identity and 60 bp alignment length^87^.

SSU rRNA gene sequence data are archived at the NCBI’s Short Read Archive (SRA accession number SRP132511; BioProject ID PRJNA433527) and GenBank (accession number MG923704 - MG923765). Metagenome-Assembled Genome data are publicly available at http://enve-omics.ce.gatech.edu/data/phenanthrene.

## Electronic supplementary material

Supplementary material
