# Supplementary material for: Anaerobic degradation of hexadecane and phenanthrene coupled to sulfate reduction by enriched consortia from northern Gulf of Mexico seafloor sediment
Source: Sci Rep. 2019 Feb 4;9:1239. doi: 10.1038/s41598-018-36567-x (PMC6361983; doi:10.1038/s41598-018-36567-x)
Supplement: Supplementary file 1 — Supplementary material [file 41598_2018_36567_MOESM1_ESM.docx]

**Anaerobic degradation of hexadecane and phenanthrene coupled to sulfate reduction by enriched consortia from the northern Gulf of Mexico seafloor sediment**

Boryoung Shin^1^, Minjae Kim^2^, Karsten Zengler^3,4^, Kuk-Jeong Chin^5^, Will A. Overholt^6^, Lisa M. Gieg^7^, Konstantinos T. Konstantinidis^2,6^, and Joel E. Kostka^1,6*^

^1^*School of Earth and Atmospheric Sciences, Georgia Institute of Technology, Atlanta, 30332, USA*

*^2^School of Civil and Environmental* *Engineering, Georgia Institute of Technology, Atlanta, 30332, USA*

^3^*Department of Pediatrics, University of California, San Diego, 92093, USA*

*^4^Center for Microbiome Innovation, University of California, San Diego, 92093, USA*

^5^*Department of Biology, Georgia State University, Atlanta, 30302, USA*

^6^*School of Biological Sciences, Georgia Institute of Technology, Atlanta, 30332, USA*

*^7^* *Department of Biological Sciences, University of Calgary, Calgary, T2N 1N4, Canada*

*joel.kostka@biology.gatech.edu

Supplemental Material

Supplemental Figure 1. The relative abundance of classes *Deltaproteobacteria*, *Anaerolineae*, and *Gammaproteobacteria* in (a) hexadecane- and (b) phenanthrene- degrading enrichment cultures under sulfate-reducing conditions. Illumina next-generation sequencing was performed using genomic DNA isolated from initial, second, third, and fourth transfers and total RNA isolated from fourth transfer. Error bars indicate standard deviation among biological replicates.

Supplemental Figure 2. Alpha-diversity based on Shannon index in (a) hexadecane- and (b) phenanthrene-degrading enrichment cultures under sulfate-reducing conditions. Error bars indicate standard deviation in triplicate for second, and third transfers, four and six replicates for fourth transfer of hexadecane- and phenanthrene-amended enrichment cultures, respectively. (c) Principal component analysis plot of beta-diversity based on the Bray-Curtis distance metric.

**(c)**

Supplemental Figure 3. Bacterial abundance as determined by qPCR of SSU rRNA genes in hexadecane-amended and phenanthrene-amended enrichment cultures under sulfate-reducing conditions.


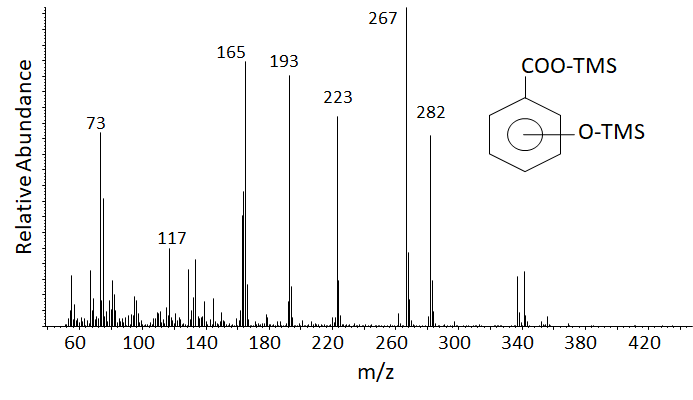


Supplemental Figure 4. Mass spectrum of a tentatively identified hydroxybenzoic acid (shown as its di-trimethylsilyl (TMS) derivative) in the phenanthrene-degrading culture.

Supplemental Figure 5. Nonpareil curves representing the complexity of PHE microbial community sampled. The circles of the curves represent the estimated average coverage at the sequencing depth/effort applied. Projected line to the right of the circle represents the expected coverage for higher sequencing efforts. Dashed lines represent 95% and 99% coverage. Curves positioned more on the right represent more sequence-diverse metagenomes compared to curves positioned on the left.

Supplemental Figure 6. Heatmap showing the amino acid identity (AAI) between genomic bins recovered. Heatmap was produced with superheat R package based on the full AAI matrix.

(A)


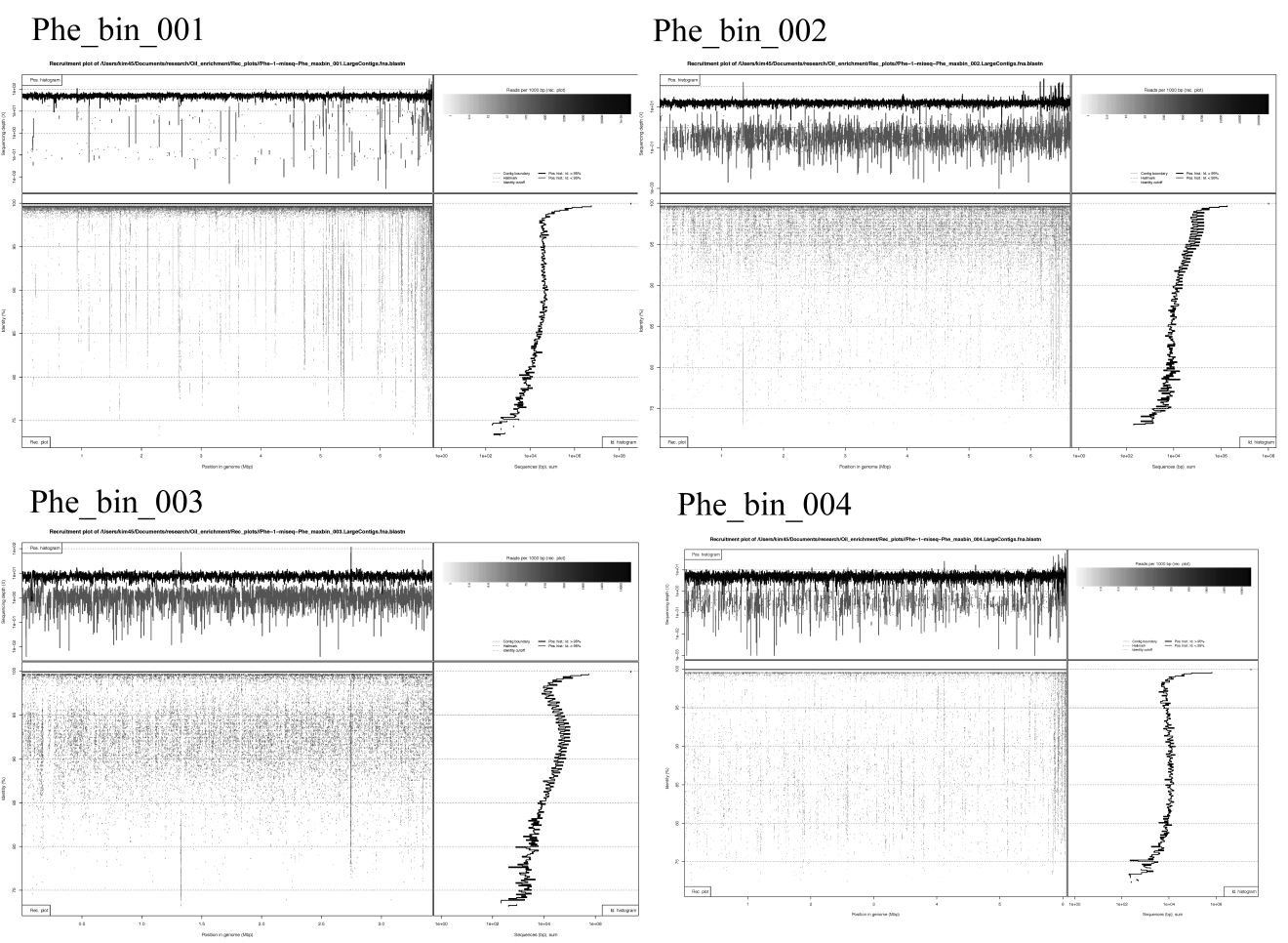


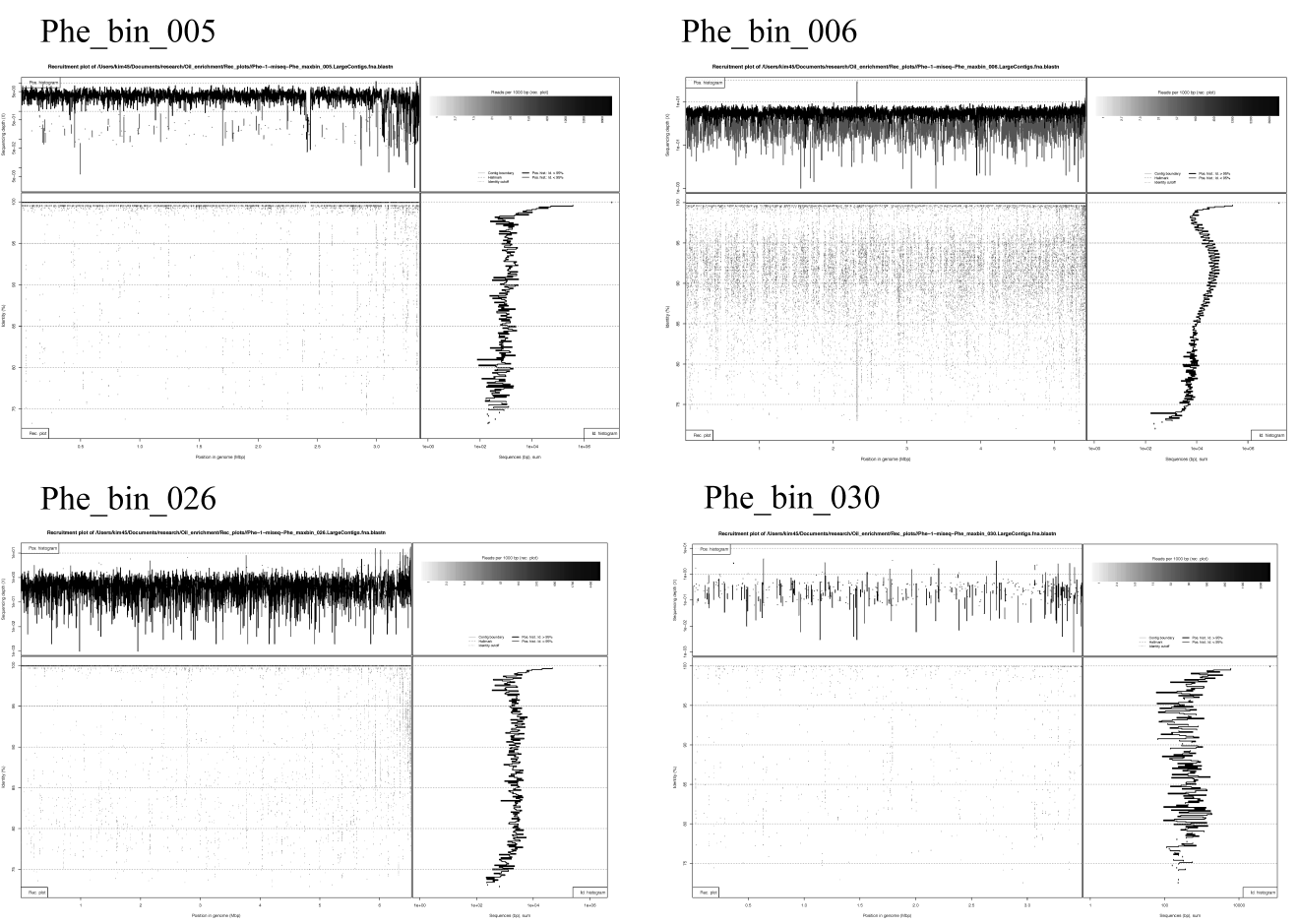


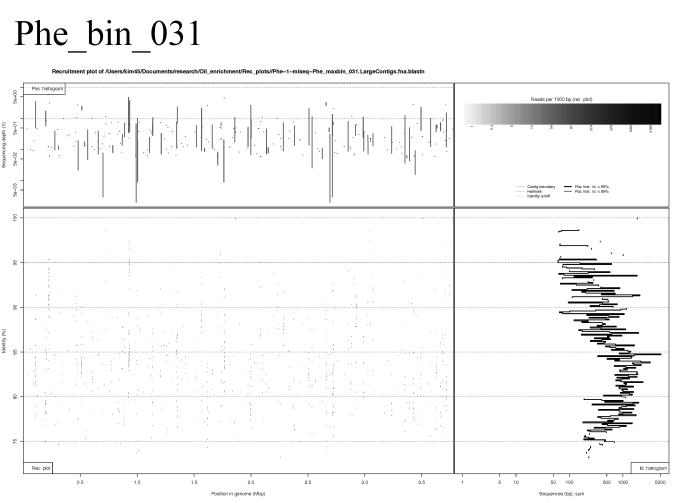


(B)


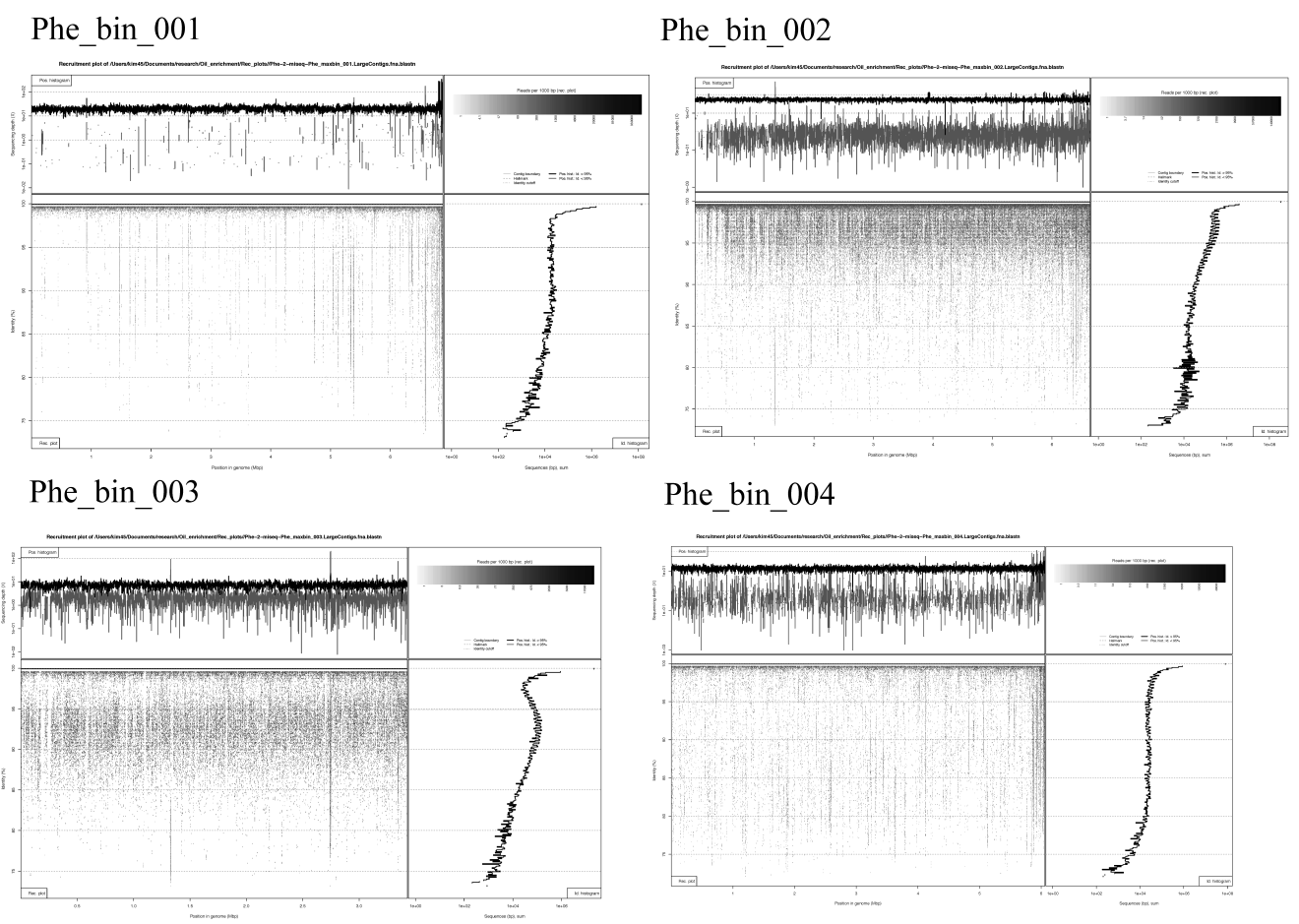


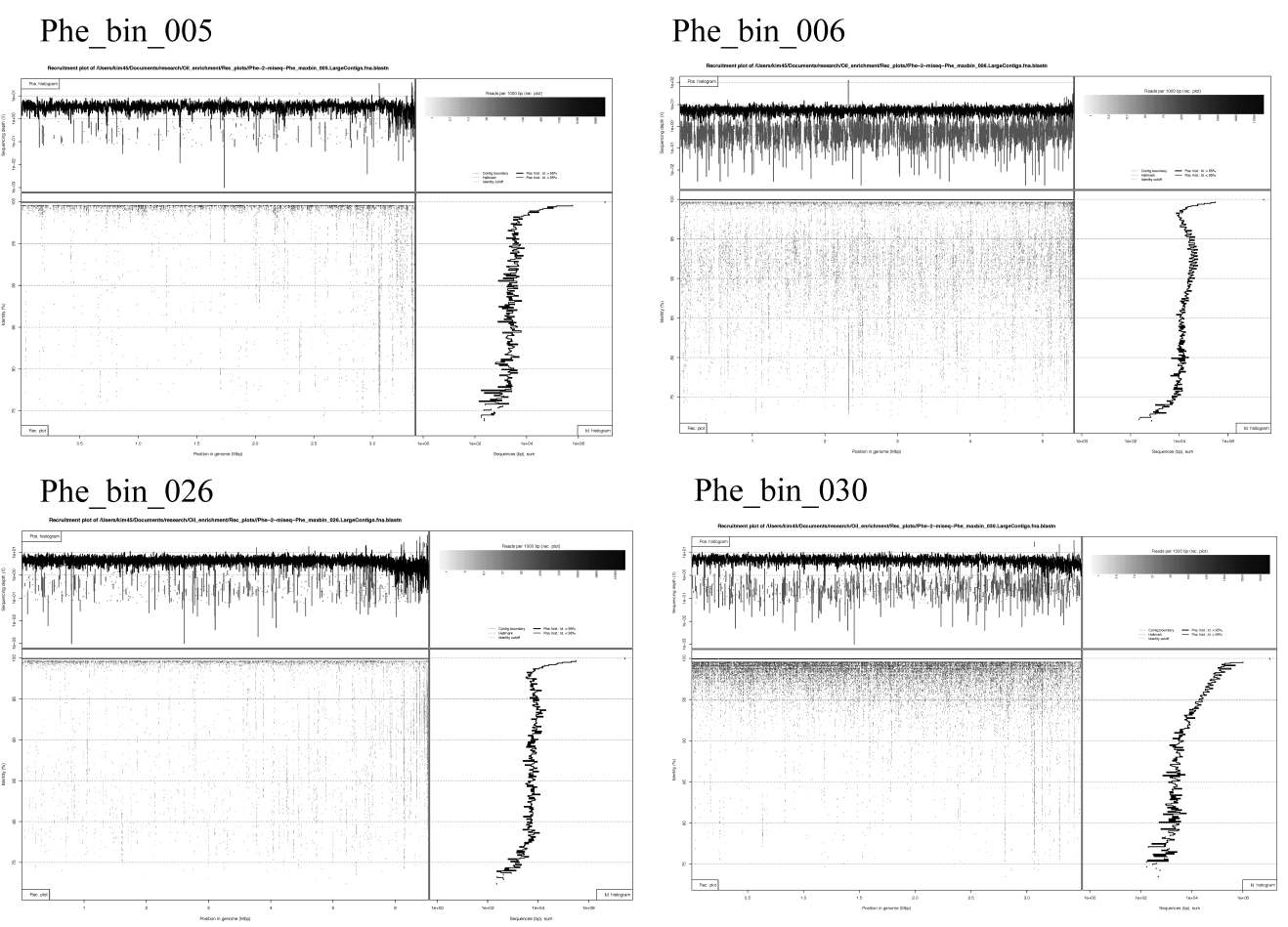


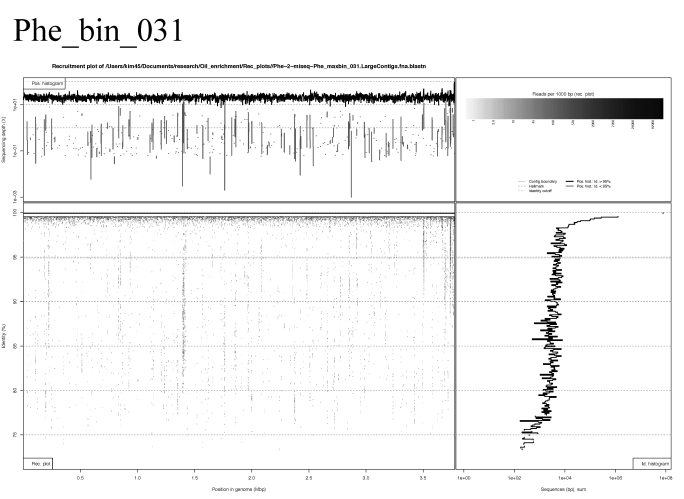


(C)


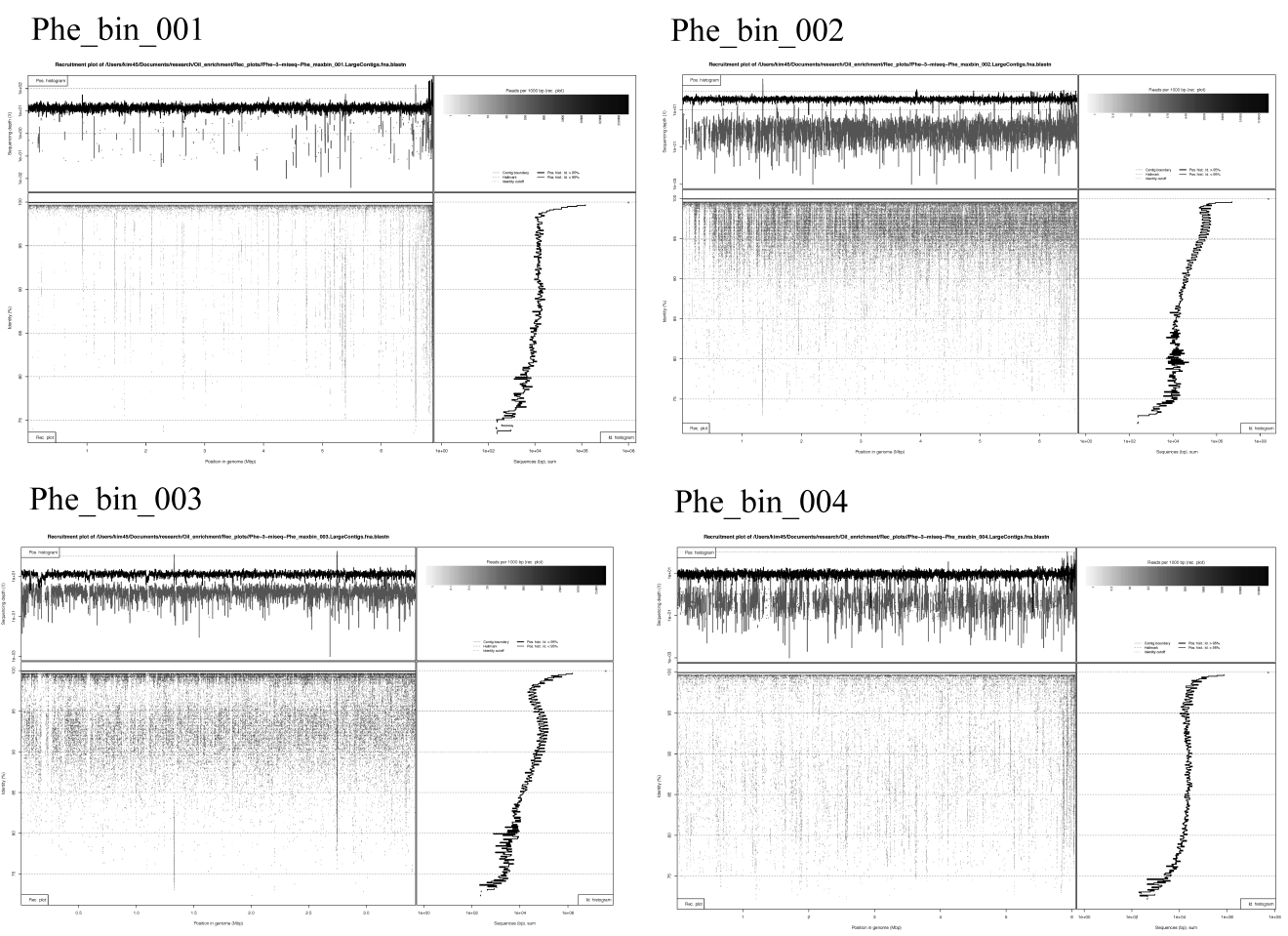


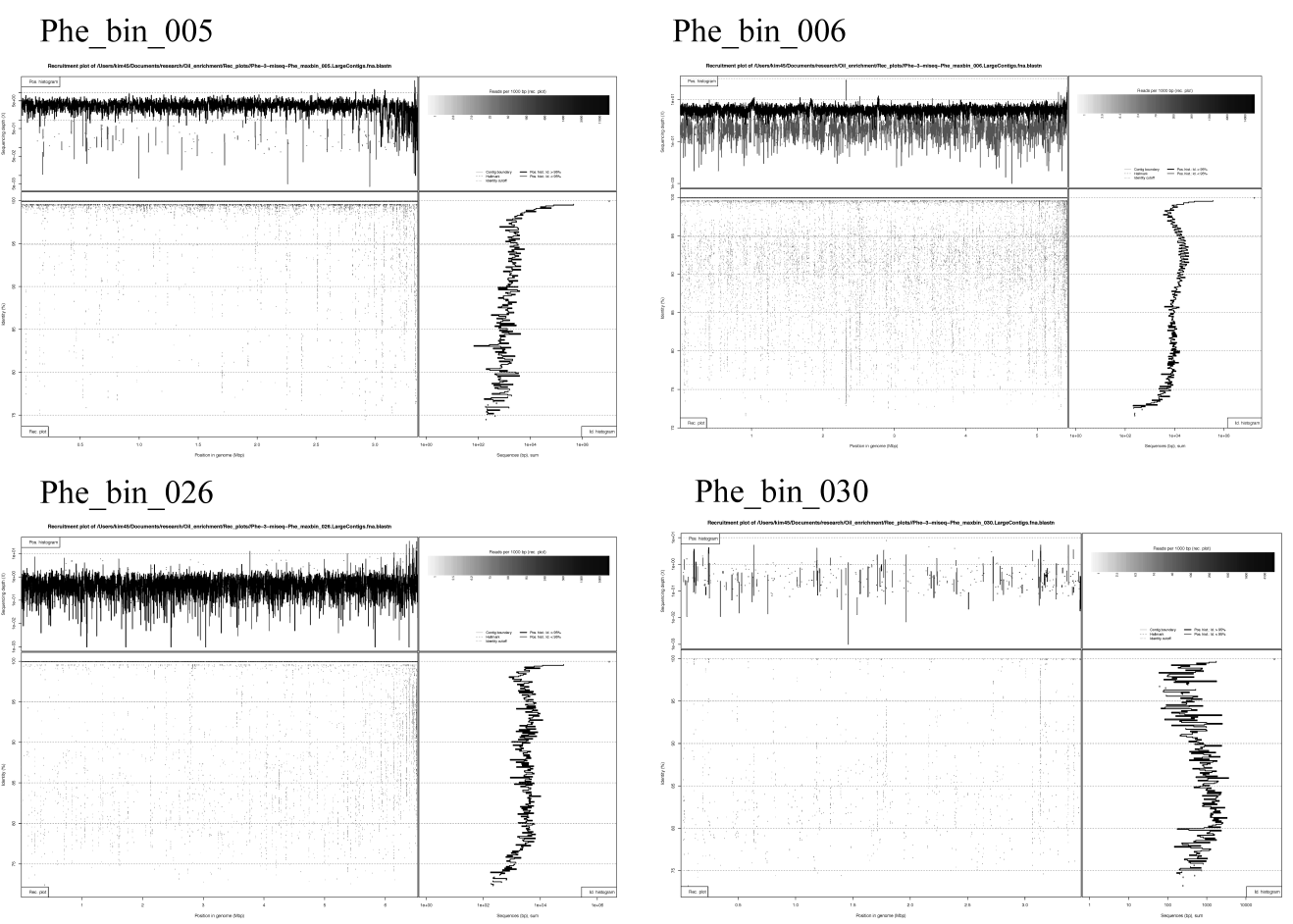


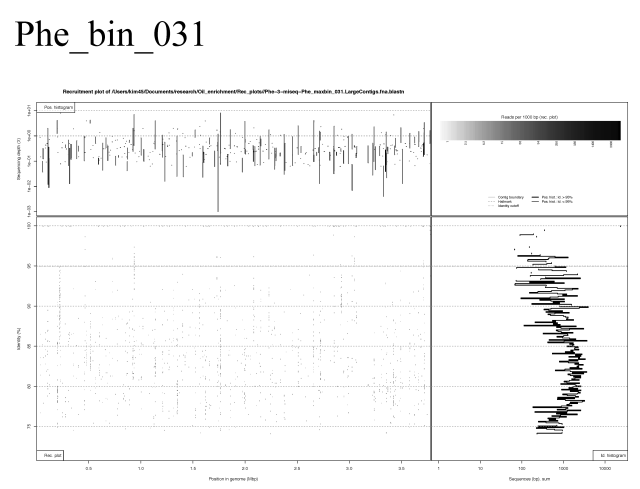


Supplemental Figure 7. Read recruitment plots of reads of PHE 4^th^ transfer metagenomes against recovered genomic bins. The fragment recruitment plot consists of four panels: (1) Bottom left panel represents the metagenomic reads recruited against the genome of bins, placed based on the location (x-axis) and percent of identity (y-axis) that they map to the genome sequence. (2) Top left panel represents sequencing depth across the reference genome sequence, i.e., number of times each nucleotide base of the reference is covered by reads, in logarithmic scale. (3) Bottom right panel represents identity histogram of mapping reads, i.e., how many bases are found at each value of nucleotide identity, in logarithmic scale. (4) Top right panel represents color scale for the number of stacked reads in bottom left panel. Panels represent metagenomic reads from: (A) Phe-1 in PHE 4^th^ transfer metagenome, (B) Phe-2 in PHE 4^th^ transfer metagenome, and (C) Phe-3 in PHE 4^th^ transfer metagenome.

Supplemental Table 1. Abundance of genome bins in PHE 4^th^ transfer metagenome.

| **Bin_ID** | **% of representation in PHE 4th** | | |
| --- | --- | --- | --- |
|  | **Phe-1** | **Phe-2** | **Phe-3** |
| Phe_bin_001 | 53.88 | 24.96 | 26.16 |
| Phe_bin_002 | 15.65 | 34.24 | 34.72 |
| Phe_bin_003 | 4.10 | 3.19 | 6.64 |
| Phe_bin_004 | 5.94 | 11.20 | 10.78 |
| Phe_bin_005 | 1.50 | 1.34 | 1.69 |
| Phe_bin_006 | 3.49 | 4.16 | 3.77 |
| Phe_bin_026 | 1.72 | 3.01 | 2.16 |
| Phe_bin_030 | NA* | 1.57 | NA |
| Phe_bin_031 | NA | 7.69 | NA |

Abundance was estimated by the sum length of reads mapping on the bin with high identity (>95% nucleotide identity). *NA: The bin is not likely to be present in metagenomes based on the read recruitment plots.

Supplemental Table 2. Protein BLAST results from recovered MAGs (> 40% Identity)

| **MAG** | **Gene** | **Putative function** | **Organism** | **Accession number** | **%Ident** | **length** |
| --- | --- | --- | --- | --- | --- | --- |
| MAG001 | AdhE | aryl-alcohol dehydrogenase | *Escherichia coli strain* K12 | WP_000301651 | 46.17 | 444 |
| MAG002 | bsdC | anaerobic 4-hydroxybenzoate carboxylase | *Bacillus subtilis* | KIX81179 | 33.75 | 480 |
| MAG002 | bsdB | anaerobic 4-hydroxybenzoate carboxylase | *Bacillus subtilis* | WP_009966530 | 48.62 | 181 |
| MAG002 | AprB | adenylsulfate reductase | *Desulfovibrio gigas* | WP_021759002 | 62.12 | 132 |
| MAG002 | AprA | adenylsulfate reductase | *Desulfovibrio gigas* | WP_021759000 | 48.17 | 654 |
| MAG002 | DsrB | dissimilatory sulfite reductase beta subunit | *Desulfovibrio vulgaris* | WP_010937710 | 64.17 | 374 |
| MAG002 | DsrA | dissimilatory sulfite reductase alpha subunit | *Desulfovibrio vulgaris* | WP_010937709 | 67.51 | 437 |
| MAG003 | AdhE | acetaldehyde dehydrogenase | *Escherichia coli strain* K12 | WP_000301651 | 44.34 | 442 |
| MAG003 | AdhE | acetaldehyde dehydrogenase | *Escherichia coli strain* K12 | WP_000301651 | 44.57 | 442 |
| MAG004 | DsrB | dissimilatory sulfite reductase beta subunit | *Desulfovibrio vulgaris* | WP_010937710 | 65.24 | 374 |
| MAG004 | DsrA | dissimilatory sulfite reductase alpha subunit | *Desulfovibrio vulgaris* | WP_010937709 | 68.65 | 437 |
| MAG004 | AprB | adenylsulfate reductase | *Desulfovibrio gigas* | WP_021759002 | 65.67 | 134 |
| MAG004 | AprA | adenylsulfate reductase | *Desulfovibrio gigas* | WP_021759000 | 46.86 | 653 |
| MAG005 | AprA | adenylsulfate reductase | *Desulfovibrio gigas* | WP_021759000 | 58.94 | 263 |
| MAG006 | AprB | adenylsulfate reductase | *Desulfovibrio gigas* | WP_021759002 | 66.67 | 135 |
| MAG006 | AprA | adenylsulfate reductase | *Desulfovibrio gigas* | WP_021759000 | 62.23 | 646 |
| MAG006 | DsrA | dissimilatory sulfite reductase alpha subunit | *Desulfovibrio vulgaris* | WP_010937709 | 76.89 | 437 |
| MAG026 | AprB | adenylsulfate reductase | *Desulfovibrio gigas* | WP_021759002 | 59.73 | 149 |
| MAG026 | AprA | adenylsulfate reductase | *Desulfovibrio gigas* | WP_021759000 | 61.36 | 660 |
| MAG026 | DsrB | dissimilatory sulfite reductase beta subunit | *Desulfovibrio vulgaris* | WP_010937710 | 74.08 | 382 |
| MAG026 | DsrA | dissimilatory sulfite reductase alpha subunit | *Desulfovibrio vulgaris* | WP_010937709 | 69.43 | 458 |
| MAG030 | AprA | adenylsulfate reductase | *Desulfovibrio gigas* | WP_021759000 | 50.81 | 372 |
| MAG030 | AprB | adenylsulfate reductase | *Desulfovibrio gigas* | WP_021759002 | 60.74 | 135 |
| MAG031 | AprA | adenylsulfate reductase | *Desulfovibrio gigas* | WP_021759000 | 80.72 | 664 |
| MAG031 | AprB | adenylsulfate reductase | *Desulfovibrio gigas* | WP_021759002 | 77.71 | 157 |
| MAG031 | DsrA | dissimilatory sulfite reductase alpha subunit | *Desulfovibrio vulgaris* | WP_010937709 | 76.77 | 439 |
| MAG031 | DsrB | dissimilatory sulfite reductase beta subunit | *Desulfovibrio vulgaris* | WP_010937710 | 74.54 | 381 |

Supplemental Figure 8. Microbial community structure analyzed by SSU rRNA genes retrieved from the metagenome of the phenanthrene-degrading enrichment culture incubated under sulfate-reducing conditions at various taxonomic levels including the (a) class , (b)family , and (c) genus level.
